# Exploring Potential Drug Targets in Multiple Cardiovascular Diseases: A Study Based on Proteome-Wide Mendelian Randomization and Colocalization Analysis

**DOI:** 10.1155/cdr/5711316

**Published:** 2025-02-21

**Authors:** Maoxia Fan, Na Li, Libin Huang, Chen Chen, Xueyan Dong, Wulin Gao

**Affiliations:** ^1^First Clinical Medical College, Shandong University of Traditional Chinese Medicine, Jinan, Shandong Province, China; ^2^Internal Medicine Department, Affiliated Hospital of Shandong University of Traditional Chinese Medicine, Jinan, Shandong Province, China; ^3^Department of Cardiology, Dongying People's Hospital (Dongying Hospital of Shandong Provincial Hospital Group), Dongying, Shandong Province, China

**Keywords:** cardiovascular diseases, colocalization analysis, drug target, Mendelian randomization, phenome-wide association study, plasma proteins

## Abstract

**Background:** Cardiovascular diseases (CVDs) encompass a group of diseases that affect the heart and/or blood vessels, making them the leading cause of global mortality. In our study, we performed proteome-wide Mendelian randomization (MR) and colocalization analyses to identify novel therapeutic protein targets for CVDs and evaluate the potential drug-related protein side effects.

**Methods:** We conducted a comprehensive proteome-wide MR study to assess the causal relationship between plasma proteins and the risk of CVDs. Summary-level data for 4907 circulating protein levels were extracted from a large-scale protein quantitative trait loci (pQTL) study involving 35,559 individuals. Additionally, genome-wide association study (GWAS) data for CVDs were extracted from the UK Biobank and the Finnish database. Colocalization analysis was utilized to identify causal variants shared between plasma proteins and CVDs. Finally, we conducted a comprehensive phenome-wide association study (PheWAS) using the R10 version of the Finnish database. This study was aimed at examining the potential drug-related protein side effects in the treatment of CVDs. A total of 2408 phenotypes were included in the analysis, categorized into 44 groups.

**Results:** The research findings indicate the following associations: (1) In coronary artery disease (CAD), the plasma proteins A4GNT, COL6A3, KLC1, CALB2, KPNA2, MSMP, and ADH1B showed a positive causal relationship (*p*‐fdr < 0.05). LAYN and GCKR exhibited a negative causal relationship (*p*‐fdr < 0.05). (2) In chronic heart failure (CHF), PLG demonstrated a positive causal relationship (*p*‐fdr < 0.05), while AZGP1 displayed a negative causal relationship (*p*‐fdr < 0.05). (3) In ischemic stroke (IS), ALDH2 exhibited a positive causal relationship (*p*‐fdr < 0.05), while PELO showed a negative causal relationship (*p*‐fdr < 0.05). (4) In Type 2 diabetes (T2DM), the plasma proteins MCL1, SVEP1, PIP4K2A, RFK, HEXIM2, ALDH2, RAB1A, APOE, ANGPTL4, JAG1, FGFR1, and MLN demonstrated a positive causal relationship (*p*‐fdr < 0.05). PTPN9, SNUPN, VAT1, COMT, CCL27, BMP7, and MSMP displayed a negative causal relationship (*p*‐fdr < 0.05). Colocalization analysis conclusively identified that AZGP1, ALDH2, APOE, JAG1, MCL1, PTPN9, PIP4K2A, SNUPN, and RAB1A share a single causal variant with CVDs (PPH3 + PPH4 > 0.8). Further phenotype-wide association studies have shown some potential side effects of these nine targets (*p*‐fdr < 0.05).

**Conclusions:** This study identifies plasma proteins with significant causal associations with CVDs, providing a more comprehensive understanding of potential therapeutic targets. These findings contribute to our knowledge of the underlying mechanisms and offer insights into potential avenues for treatment.

## 1. Introduction

Cardiovascular diseases (CVDs) encompass a cluster of ailments that affect the heart and/or blood vessels, including hypertension, coronary artery disease (CAD), atrial fibrillation (AF), heart failure, and stroke. As of 2019 estimates, CVDs accounted for approximately 179 million global fatalities, making them the leading cause of death worldwide [1, 2]. Projections indicate that by 2030, the number of deaths related to CVDs could surpass 236 million [3]. As the global population ages, the socioeconomic burden resulting from CVDs has been steadily increasing. The etiology of CVDs is complex, often developing over several decades before manifesting as overt symptomatic events. Early intervention is crucial in reducing the incidence and mortality rates associated with CVD, which would have profound implications for the burden on public health [4]. By enhancing our comprehension of the causal relationships among various risk factors, especially at the microscopic and molecular levels, novel drug targets can be discerned through the investigation of specific molecular pathways or biomarkers. For instance, pharmaceutical interventions targeting specific proteins, genes, or signaling pathways can modulate the progression of CVDs thereby refining preventive strategies. Consequently, the identification of fresh drug targets for therapeutic interventions in CVDs stands as a pivotal avenue in contemporary medical research.

Plasma proteins play crucial roles in various biological processes, including signal transduction, transport, growth, repair, and immune defense. Dysregulation of these proteins is commonly observed in various diseases, making them important targets for drug development [5]. Recently, Mendelian randomization (MR) analysis has emerged as a widespread tool for repurposing existing drugs and discovering new therapeutic targets [6, 7]. Genome-wide association study (GWAS) has identified specific single-nucleotide polymorphisms (SNPs) that regulate protein expression on the chromosome. These SNPs, which are positively associated with protein abundance, are referred to as protein quantitative trait loci (pQTL) [8]. Integrating GWAS and pQTL summary statistics is aimed at identifying potential disease-relevant proteins. In particular, phenome-wide association study (PheWAS) allows for the detection of protein-coding genes that are functionally altered and associated with phenotypes [9]. MR analysis utilizes these pQTLs as instrumental variables to explore potential causal relationships between exposures and outcomes, aiding in the identification of drug targets and biomarkers [10, 11]. Compared to observational studies, MR analysis helps mitigate the impact of confounding factors, thereby enhancing the reliable assessment of causal relationships [12].

**Figure 1:**
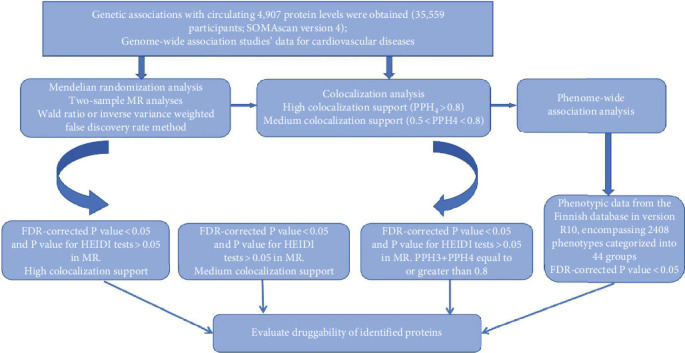
Flowchart of the study design.

The recent large-scale study investigating the genetic associations with circulating protein levels has provided a unique opportunity to comprehensively examine the causal impact of circulating proteins on CVDs. Consequently, we conducted an extensive proteome-wide MR study to preliminarily ascertain the associations between proteins and diseases. Subsequently, we performed colocalization analysis to validate the reliability of our research findings. Lastly, we evaluated the potential adverse reactions of drug proteins used in CVD treatment through a PheWAS. The study's comprehensive design is depicted in Figure 1.

## 2. Materials and Methods

### 2.1. Plasma pQTLs

From a large-scale study on pQTLs involving 35,559 individuals of Icelandic descent, we extracted GWAS data for 4907 plasma proteins [13]. pQTLs are genomic loci that are often in close proximity to the corresponding genes that are associated with specific proteins. In fact, a pQTL located near its homologous gene is referred to as a “cis-pQTL,” suggesting that this pQTL exerts its influence through the homologous gene. Conversely, if a pQTL is situated far from its homologous gene or on a different chromosome, it is termed a “trans-pQTL,” hypothesizing that it operates through an intermediary gene [8]. Various studies have employed different distance cutoff methods to distinguish cis-pQTLs from trans-pQTLs within the chromosome, with commonly used thresholds of 500 kb or 1000 kb [14, 15]. In our comprehensive proteome-wide study targeting drug targets, we employed pQTLs as instrumental variables, applying specific selection criteria. These criteria are outlined as follows: (1) cis-acting pQTLs: SNPs in the vicinity of gene regions within ±1 Mb were considered; (2) genome-wide significance threshold: SNPs highly correlated with plasma proteins were identified based on a significance threshold of *p* < 5 × 10^−8^; (3) to ensure the inclusion of independent SNPs and mitigate the impact of linkage disequilibrium (LD) on the results, we set a LD parameter (*r*^2^) of 0.001 and a genetic distance of 10,000 kb; and (4) exclusion of weak instrumental variable bias: We applied an *F*-value threshold greater than 10 to eliminate weak instrumental variables [16–18].

### 2.2. GWAS Statistics of CVDs

This study utilized summary data from GWAS for conducting MR analysis. The CVDs included in the analysis encompassed CAD, essential hypertension (EH), AF, chronic heart failure (CHF), ischemic stroke (IS), Type 2 diabetes (T2DM), atherosclerosis (AS), venous thromboembolism (VTE), and peripheral artery disease (PAD).

For a detailed description of the data sources, please refer to Table 1.

### 2.3. MR Analysis

In this study, we conducted a two-sample MR analysis using 4907 plasma proteins as exposures and CVDs as outcomes. The selection criteria for pQTLs remained consistent with the criteria outlined earlier. For the MR analysis, we utilized the R package “TwoSampleMR” (Version 0.5.6). When a specific protein had only one available SNP, we employed the Wald ratio method. Conversely, if two or more SNPs were available, we applied the inverse variance weighted (IVW) method [19]. To account for multiple comparisons, we employed the false discovery rate (FDR) method for *p* value correction. *p*‐fdr values below 0.05 were considered statistically significant. Further sensitivity analyses were performed using the Cochran *Q*-test and the MR-Egger intercept test to assess the heterogeneity and horizontal pleiotropy of the MR results [20, 21]. For MR analyses where the instrumental variables contain more than two SNPs, the MR results were considered robust if the weighted median, the weighted mode, the simple mode, and the MR-Egger model yield MR estimates consistent with the IVW direction. The MR-Egger result was only considered if the MR estimator was multidirectional. The Steiger test was applied to explore whether there was a reverse causal relationship between exposure and outcome [22].

### 2.4. Colocalization Analysis

We performed colocalization analysis using the colocal R package to assess whether the observed associations between the identified positive proteins and various CVDs were driven by LD. Colocalization analysis is aimed at confirming the presence of shared causal genetic variation between the exposure and outcome, further validating the findings of the MR study. For the positively associated proteins identified in the MR analysis, we conducted colocalization analysis by examining the SNPs within ±1 Mb of the upstream and downstream regions of the genes (cis-pQTLs) corresponding to these proteins and their colocalization with CVDs [23]. The colocalization analysis involves five hypotheses: PPH0: Phenotype 1 and Phenotype 2 are not significantly associated with all SNP loci in a given genomic region; PPH1: Phenotype 1 is significantly associated with a SNP locus in a genomic region; PPH2: Phenotype 2 is significantly associated with a SNP locus in a genomic region; PPH3: Phenotype 1 and Phenotype 2 are significantly associated with SNP loci in a given genomic region but are driven by different causal variant loci; PPH4: Phenotype 1 and Phenotype 2 are significantly associated with SNP loci in a genomic region and driven by the same causal variant locus [24]. Proteins with high support for colocalization evidence (PPH4 > 0.8) are considered primary target proteins. Proteins with moderate support for colocalization evidence (0.5 < PPH4 < 0.8) are classified as secondary target proteins. The remaining proteins are considered tertiary target proteins [25]. Due to limited power in the colocalization analysis, we focused our examination on genes with a combined posterior probability of association (PPH3 + PPH4) equal to or greater than 0.8. PPH3 + PPH4: Phenotype 1 and Phenotype 2 are significantly associated with SNP loci in a genomic region [24].

### 2.5. PheWAS

PheWAS, also known as reverse GWAS, is a method utilized to explore associations between SNPs or phenotypes and a wide array of phenotypes spanning the entire phenome [26, 27]. This approach is particularly valuable for investigating potential side effects related to drug targets [24]. In this study, the exposure was derived from plasma proteins exhibiting positive MR results, and the screening criteria for instrumental variables remained consistent with those described previously. The outcome involved obtaining phenotypic data from the Finnish database in Version R10, encompassing 2408 phenotypes categorized into 44 groups. This extensive dataset was employed for phenome-wide MR analysis. A *p*‐fdr value below 0.05 was considered statistically significant.

## 3. Results

### 3.1. Plasma Proteins Related to CVDs

In this study, after strictly adhering to the instrumental variable selection criteria, no protein targets were found to have a causal relationship with EH, AF, AS, VTE, and PAD among the 4907 plasma proteins tested (*p*‐fdr > 0.05).

**Figure 2:**
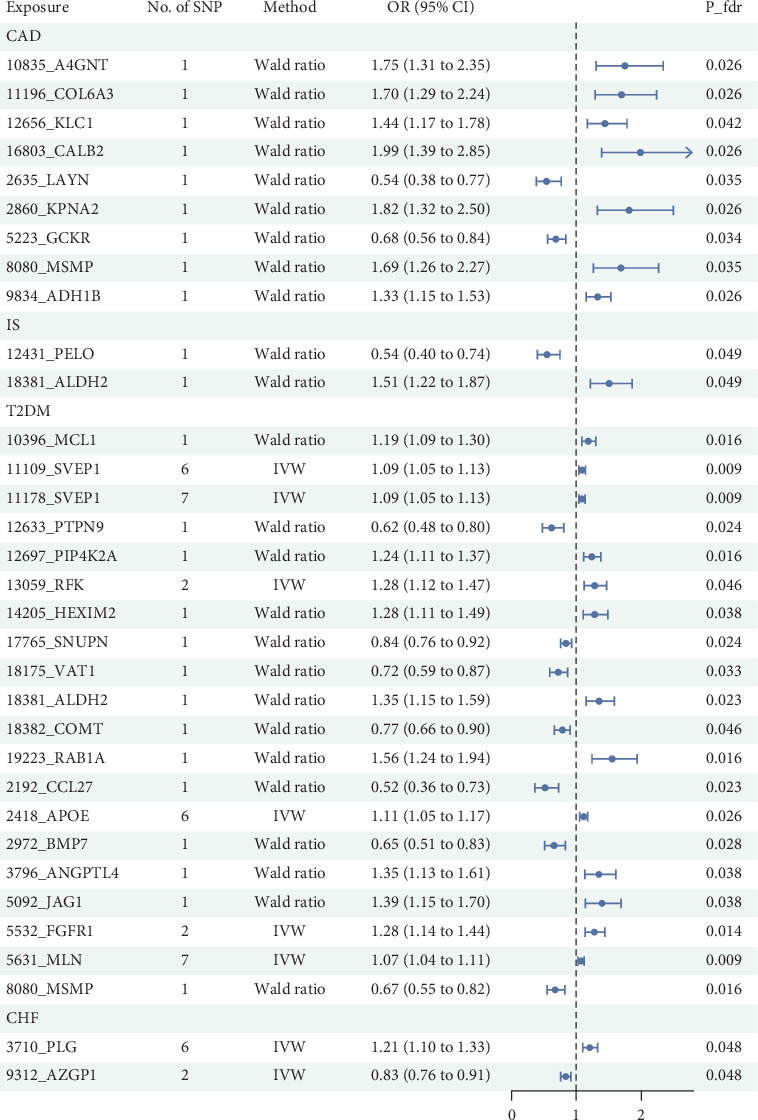
Forest plot of the MR results. Note: CAD: coronary artery disease, IS: ischemic stroke, T2DM: Type 2 diabetes, CHF: chronic heart failure, SNP: single-nucleotide polymorphism, IVW: inverse variance weighted, MR: Mendelian randomization.

**Figure 3:**
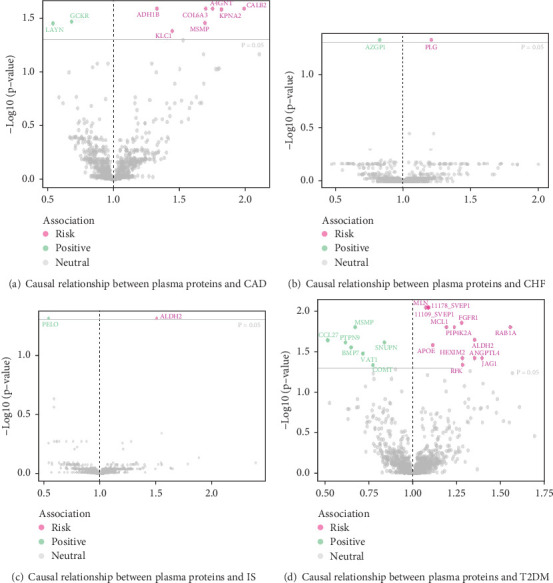
Volcano plot of MR results: causal relationship between plasma proteins and CVDs. Note: CAD: coronary artery disease, CHF: chronic heart failure, IS: ischemic stroke, T2DM: Type 2 diabetes, CVDs: cardiovascular diseases, MR: Mendelian randomization.

It is worth noting that among the 4907 plasma proteins tested, the MR analysis based on IVW or Wald ratio results (*p*‐fdr < 0.05) revealed positive protein associations with CAD, CHF, T2DM, and IS. (1) In the case of CAD, the following proteins showed a positive causal relationship, indicating a potential association with an increased risk of CAD: A4GNT (alpha-1,4-N-acetylglucosaminyltransferase) [OR = 1.75, 95% CI (1.31 to 2.35), *p*‐fdr = 0.026], COL6A3 (collagen, type VI, alpha 3) [OR = 1.70, 95% CI (1.29–2.24), *p*‐fdr = 0.026], KLC1 (kinesin 2 60–70 kDa protein) [OR = 1.44, 95% CI (1.17–1.78), *p*‐fdr = 0.042], CALB2 (calbindin 2 protein) [OR = 1.99, 95% CI (1.39–2.85), *p*‐fdr = 0.026], KPNA2 (karyopherin alpha 2 protein) [OR = 1.82, 95% CI (1.32–2.50), *p*‐fdr = 0.026], MSMP (microseminoprotein, prostate-associated protein) [OR = 1.69, 95% CI (1.26–2.27), *p*‐fdr = 0.035], and ADH1B (alcohol dehydrogenase 2) [OR = 1.33, 95% CI (1.15–1.53), *p*‐fdr = 0.026]; LAYN (layilin protein) [OR = 0.54, 95% CI (0.38–0.77), *p*‐fdr = 0.035] and GCKR (glucokinase regulatory protein) [OR = 0.68, 95% CI (0.56–0.84), *p*‐fdr = 0.034] showed a negative causal relationship, suggesting that they may act as protective factors in preventing the occurrence of CAD. (2) In the case of CHF, the protein PLG (plasminogen) [OR = 1.21, 95% CI (1.10–1.33), *p*‐fdr = 0.048] showed a positive causal relationship, suggesting a potential association with an increased risk of CHF; AZGP1 (alpha-2-glycoprotein 1, zinc-binding protein) [OR = 0.83, 95% CI (0.76–0.91), *p*‐fdr = 0.048] exhibited a negative causal relationship, indicating that it may act as a protective protein against CHF. (3) In the case of IS, the protein ALDH2 (acetaldehyde dehydrogenase 2) [OR = 1.51, 95% CI (1.22–1.87), *p*‐fdr = 0.049] showed a positive causal relationship, suggesting a potential association with an increased risk of IS; PELO (pelota protein) [OR = 0.54, 95% CI (0.40–0.74), *p*‐fdr = 0.049] exhibited a negative causal relationship, indicating that it may act as a protective factor in preventing the occurrence of IS. (4) In the case of T2DM, the following plasma proteins showed a positive causal relationship, indicating a potential association with an increased risk of T2DM: MCL1 (myeloid cell leukemia sequence 1 (BCL2-related) protein) [OR = 1.19, 95% CI (1.09–1.30), *p*‐fdr = 0.016], SVEP1 (pentraxin domain-containing protein 1) [OR = 1.09, 95% CI (1.05–1.13), *p*‐fdr = 0.009], PIP4K2A (phosphatidylinositol-5-phosphate 4-kinase, type II, alpha protein) [OR = 1.24, 95% CI (1.11–1.37), *p*‐fdr = 0.016], RFK (riboflavin kinase) [OR = 1.28, 95% CI (1.12–1.47), *p*‐fdr = 0.046], HEXIM2 (hexamthylene bis-acetamide-inducible 2 protein) [OR = 1.28, 95% CI (1.11–1.49), *p*‐fdr = 0.038], ALDH2 [OR = 1.35, 95% CI (1.15–1.59), *p*‐fdr = 0.023], RAB1A (RAB1A, member RAS oncogene family protein) [OR = 1.56, 95% CI (1.24–1.94), *p*‐fdr = 0.016], APOE (apolipoprotein E) [OR = 1.11, 95% CI (1.05–1.17), *p*‐fdr = 0.026], ANGPTL4 (angiopoietin-like 4 protein) [OR = 1.35, 95% CI (1.13–1.61), *p*‐fdr = 0.038], JAG1 (jagged 1 protein) [OR = 1.39, 95% CI (1.15–1.70), *p*‐fdr = 0.038], FGFR1 (fibroblast growth factor receptor-1c) [OR = 1.28, 95% CI (1.14–1.44), *p*‐fdr = 0.014], and MLN (myoregulin) [OR = 1.07, 95% CI (1.04–1.11), *p*‐fdr = 0.009]. The following plasma proteins exhibited a negative causal relationship, suggesting that their presence may reduce the risk of developing T2DM: PTPN9 (protein tyrosine phosphatase, nonreceptor type 9) [OR = 0.62, 95% CI (0.48–0.80), *p*‐fdr = 0.024], SNUPN (snurportin 1 protein) [OR = 0.84, 95% CI (0.76–0.92), *p*‐fdr = 0.024], VAT1 (vesicle amine transport 1 protein) [OR = 0.72, 95% CI (0.59–0.87), *p*‐fdr = 0.033], COMT (catechol-O-methyltransferase protein) [OR = 0.77, 95% CI (0.66–0.90), *p*‐fdr = 0.046], CCL27 (chemokine (C-C motif) ligand 27 protein) [OR = 0.52, 95% CI (0.36–0.73), *p*‐fdr = 0.023], BMP7 (bone morphogenetic protein 7) [OR = 0.65, 95% CI (0.51–0.83), *p*‐fdr = 0.028], and MSMP [OR = 0.67, 95% CI (0.55–0.82), *p*‐fdr = 0.016]. For details, see Figures 2 and 3. Relevant SNP information involved in the MR analysis is provided in Supporting Information 1. The results of MR analysis of plasma proteins and CVDs are presented in Supporting Information 2.

### 3.2. Colocalization Analysis

For the selected plasma proteins, we conducted gene colocalization analysis within a ±1 Mb range upstream and downstream of their respective genes to explore their potential associations with CVDs. (1) In the colocalization analysis of CAD, it was observed that for these nine plasma proteins (LAYN, KPNA2, GCKR, MSMP, ADH1B, A4GNT, COL6A3, KLC1, CALB2), there may not be shared causal variants in the region associated with CAD (PPH3 + PPH4 < 0.8). (2) In the colocalization analysis of CHF, the results indicate that AZGP1 may share a causal variant in the region associated with CHF (PPH3 + PPH4 > 0.8), while PLG may not have shared causal variants in the same region (PPH3 + PPH4 < 0.8). (3) In the colocalization analysis of IS, the results indicate that ALDH2 may share a causal variant in the region associated with IS (PPH3 + PPH4 > 0.8), while PELO may not have shared causal variants in the same region (PPH3 + PPH4 < 0.8). (4) In the colocalization analysis of T2DM, the results indicate that APOE, JAG1, MCL1, PTPN9, PIP4K2A, SNUPN, and RAB1A may share a causal variant in the region associated with T2DM (PPH3 + PPH4 > 0.8). However, CCL27, BMP7, ANGPTL4, FGFR1, MLN, MSMP, SVEP1, RFK, HEXIM2, VAT1, ALDH2, and COMT may not have shared causal variants in the same region associated with T2DM (PPH3 + PPH4 < 0.8). These findings suggest that these nine plasma proteins may serve as potential therapeutic targets for CVDs. Results of gene colocalization analysis between plasma proteins and CVDs are provided in Table 2.

### 3.3. Phenome-Wide Association Analysis for Nine Plasma Proteins Linked to CVDs

**Figure 4:**
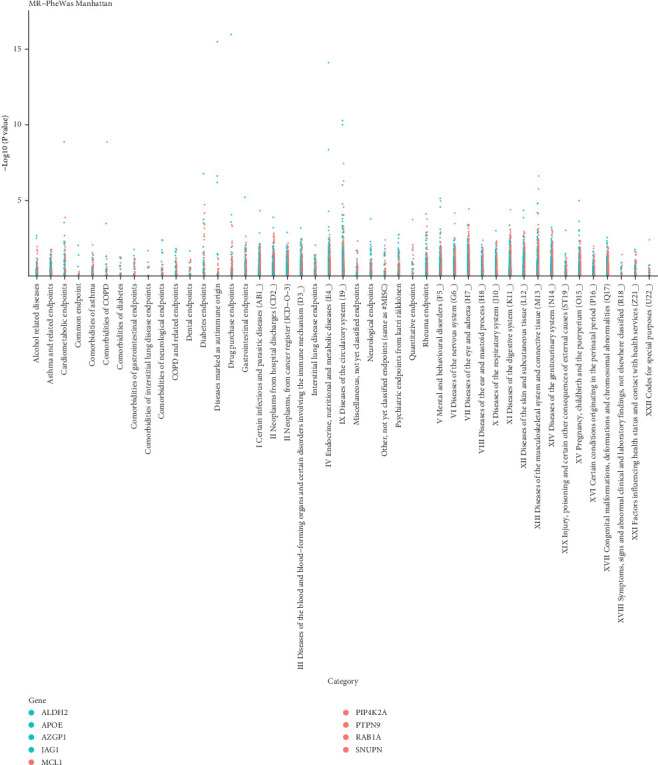
Manhattan plot of result of PheWAS analysis of associations between nine CVD-associated plasma proteins and other disease outcomes. Note: PheWAS: phenome-wide association study, CVDs: cardiovascular diseases.

To assess the potential beneficial or harmful effects of the nine plasma proteins associated with CVDs on other phenotypes, we conducted a comprehensive phenome-wide association analysis. We screened 2408 phenotypes from 44 categories in the Finnish database (R10 version), which comprised 412,181 individuals. We observed significant causal relationships (*p*‐fdr < 0.05) between PTPN9 and three phenotypes in three categories, between PIP4K2A and one phenotype in one category, between SNUPN and six phenotypes in five categories, between ALDH2 and nineteen phenotypes in eleven categories, between RAB1A and six phenotypes in four categories, between APOE and one phenotype in one category, between JAG1 and one phenotype in one category, and between AZGP1 and two phenotypes in one category. These phenotypes may serve as potential treatment targets or indicate harmful effects on the target proteins. See further details in Figure 4. Results of PheWAS analysis of associations between nine CVD-associated plasma proteins and other disease outcomes are provided in Supporting Information 3.

## 4. Discussion

In the past few decades, advancements in both pharmacological and nonpharmacological treatments for CVDs have led to a significant reduction in cardiovascular mortality rates. However, the global burden of CVDs continues to rise. It is imperative to identify potential therapeutic targets for CVDs and develop new drugs for the treatment of patients. In this study, we conducted a comprehensive protein-centric systems biology integration analysis by sequentially using MR, colocalization analysis, and PWAS. This approach allowed us to identify plasma proteins associated with CVDs and explore their potential causal relationships with relevant CVDs. The results of this study indicate that there is no MR analysis evidence suggesting a potential causal relationship between 4907 plasma proteins and the genetic susceptibility to EH, AF, AS, VTE, and PAD risk. After identifying potential LD effects through colocalization analysis, there is no longer a potential causal relationship between plasma proteins and CAD. However, there is strong evidence supporting the following associations: (1) Higher genetic prediction levels of AZGP1 are negatively associated with CHF risk; (2) higher genetic prediction levels of ALDH2 are positively associated with IS risk; and (3) higher genetic prediction levels of APOE, JAG1, MCL1, PIP4K2A, and RAB1A are positively associated with T2DM risk, and higher genetic prediction levels of PTPN9 and SNUPN are inversely associated with T2DM risk.

AZGP1, also commonly abbreviated as ZAG, is a secreted 43-kDa protein expressed in various epithelial tissues and adipocytes. It circulates in high concentrations in human blood. AZGP1 is considered a regulatory factor in metabolic functions, blood pressure regulation, neurologic disorders, and CVDs [28, 29]. Studies have demonstrated that elevated levels of AZGP1 exert a protective effect on the heart [30–32], making it a cardiovascular protective factor capable of reducing the risk of CVDs such as CHF. Some existing therapeutic approaches can impact the expression of AZGP1. Weight management medications, such as those for weight loss or appetite regulation, may influence the expression of AZGP1. These medications could affect the expression levels of the AZGP1 protein by altering lipid metabolic pathways or appetite regulation pathways. Insulin-regulating medications, such as insulin or insulin sensitizers, might also influence the expression of AZGP1 due to its association with sugar metabolism. Hormone therapies, including medications of a hormonal nature, could affect protein expression in adipose tissue since AZGP1 is expressed in adipocytes, and certain hormones may impact the functionality and metabolism of adipocytes. Metabolic-regulating medications, such as those aimed at regulating fat or sugar metabolism, could potentially affect the expression levels of AZGP1. Exercise therapy: Moderate exercise can influence lipid metabolism and protein expression; therefore, exercise therapy could potentially impact the expression of AZGP1.

ALDH2 is a mitochondrial enzyme involved in the detoxification of exogenous and endogenous aldehydes [33]. Increasing epidemiological evidence suggests a close association between ALDH2 gene polymorphisms and increased cardiovascular risk factors and stroke incidence. Meta-analyses have also indicated that ALDH2 may serve as a risk factor for IS. Consequently, targeting ALDH2 for therapeutic purposes may represent a promising approach to prevent stroke-related damage [34, 35]. Some existing therapeutic methods can influence its expression. Alcohol consumption: Alcohol intake can increase endogenous acetaldehyde production, thereby increasing the demand for ALDH2 activity. Long-term excessive alcohol consumption may have adverse effects on the expression and activity of ALDH2. Drug interactions: Certain medications can affect the activity of ALDH2. For instance, some drugs might interfere with ALDH2 enzyme activity or modulate its expression, thus impacting acetaldehyde metabolism and detoxification. Oxidative stress: Oxidative stress is a condition of intracellular imbalance that can lead to mitochondrial dysfunction, thereby affecting ALDH2 activity. Antioxidant therapy may help alleviate the detrimental effects of oxidative stress on ALDH2. Metabolic disease treatment: Certain metabolic disorders, such as diabetes, can influence mitochondrial function and the expression of related enzymes. Treating these diseases may affect the function of ALDH2. Nutritional supplementation: Some nutrients and vitamins are crucial for maintaining mitochondrial function and enzyme activity. Proper nutritional supplementation may aid in sustaining the normal function of ALDH2.

APOE is present in circulation, associated with chylomicrons, very low-density lipoprotein (VLDL), and high-density lipoprotein (HDL), with typical concentrations ranging from 20 to 60 mg/L [36]. It has been reported that APOE isoforms are important genetic markers for dyslipidemia [37]. Previous studies have indicated that APOE is among the most likely candidate genes associated with CAD in patients with T2DM [38]. Meta-analyses have shown that APOE is positively associated with an increased risk of developing T2DM, suggesting its potential role as a risk factor for T2DM incidence [39]. Here are some potential therapeutic methods that may affect the expression or function of ApoE. Lipid-lowering medications: Drugs designed to reduce lipid levels, such as statins and fibrate drugs, could impact the expression and function of ApoE through various pathways. These medications might regulate cholesterol metabolic pathways, thus influencing the role of ApoE. Antioxidants: The use of antioxidants may help alleviate the detrimental effects of oxidative stress on ApoE function. Oxidative stress could lead to conformational changes or degradation of the ApoE protein. Exercise therapy: Moderate exercise can affect cholesterol metabolism and the expression of related lipoproteins, including ApoE. Exercise might influence the function of ApoE by modulating metabolic pathways. Dietary control: Controlling the fat and cholesterol content in the diet could potentially impact the expression and function of ApoE. Specific dietary patterns might help regulate cholesterol metabolic pathways and influence the role of the ApoE protein. Gene therapy: Therapeutic approaches targeting the ApoE gene could directly affect the expression levels or function of ApoE. For example, gene editing technologies could be used to modulate the expression of ApoE. Drug interactions: Certain medications could interfere with the metabolism or function of ApoE, thereby affecting cholesterol metabolism and the risk of CVDs.

JAG1 is one of the five cell surface ligands that primarily functions in the highly conserved Notch signaling pathway. In the heart, in situ hybridization studies have demonstrated the expression of JAG1 in the vascular structures of mammalian hearts, which is associated with cardiovascular phenotypes observed in Alagille syndrome (ALGS) [40]. Furthermore, cytokines play a crucial role in inflammatory diseases such as diabetes and are implicated in the expression and signaling pathways of JAG1 and Notch [41]. The gene expression levels of JAG1 are closely associated with human diabetes, increasing the risk of developing the disease [42]. Some existing therapeutic methods can influence the expression of JAG1. Notch signaling pathway modulators: Medications or therapeutic approaches might directly regulate the activity of the Notch signaling pathway, thereby affecting the expression of JAG1. These medications could act as activators or inhibitors of the Notch signaling pathway. Chemotherapy drugs: Some chemotherapy medications could impact the activity of the Notch signaling pathway, consequently affecting the expression of JAG1. This influence could be significant for tumor treatment and cell proliferation. Immunomodulatory therapy: Immunomodulatory treatments might indirectly affect the expression of JAG1 by regulating the activity of immune cells and signaling pathways. This impact could be crucial for the treatment of immune-related diseases. Anti-inflammatory drugs: Anti-inflammatory medications could influence the expression of JAG1 by modulating inflammatory responses and cellular signaling pathways. This effect could be important for the treatment of inflammatory conditions. Stem cell therapy: Stem cell therapy could impact the expression of JAG1 by modulating cellular signaling pathways and cell fate determination. This therapeutic approach could be significant for tissue regeneration, injury repair, and other applications. Gene therapy: Treatment approaches targeting the JAG1 gene could directly affect the expression levels or function of JAG1, for example, by regulating JAG1 expression through gene editing technologies.

MCL1 is an antiapoptotic member of the B-cell lymphoma 2 (BCL2) family characterized by its short half-life and its role as a rapid sensor for regulating cell death and other related processes, including cell cycle progression and mitochondrial homeostasis [43]. Although this experimental study does not directly indicate an increased risk of developing T2DM associated with MCL1, it suggests that targeting the MCL1 gene could be beneficial in preventing the progression of diabetes, potentially offering new avenues for the treatment of diabetes [44]. Some existing therapeutic methods can influence its expression. Cancer drug therapy: Chemotherapy and targeted therapy drugs may impact the expression of MCL1 by modulating the cell apoptosis pathway. Some medications might promote tumor cell apoptosis by inhibiting MCL1 expression or regulating its function. Drugs targeting MCL1: In recent years, researchers have developed drugs directly targeting MCL1, which could affect the process of cell apoptosis by modulating the expression or function of MCL1. Immunotherapy: Immunotherapy may activate immune cells to identify and destroy tumor cells, with some mechanisms potentially involving the regulation of MCL1 expression in the apoptosis pathway. Radiation therapy: Radiation therapy, a common cancer treatment method, can induce cell apoptosis by causing DNA damage. MCL1 may play a crucial role in regulating the process of cell apoptosis. Protein stability modulators: Some drugs may influence protein stability, including affecting the half-life of MCL1. These medications could impact the expression levels of MCL1 by regulating its stability. Nutritional intervention: Proper nutritional intervention might affect cellular metabolic pathways and the expression of apoptosis regulatory factors, including MCL1.

PIP4K2A regulates lipid droplet formation, phospholipid metabolism, and cholesterol transport by modulating the steady-state levels of phosphatidylinositol 4,5-bisphosphate (PI(4,5)P2) on the peroxisome [45]. Additionally, PIP4K2A exhibits distinct catalytic and noncatalytic functions in controlling cellular metabolism, and the loss of catalytic-independent functions of PIP4Ks forms the basis for enhanced insulin signaling [46]. These findings indirectly suggest that the presence of the PIP4K2A gene increases the risk of developing diabetes. Some existing therapeutic methods can influence its expression. PI3K inhibitors: PI3K (phosphoinositide 3-kinase) is a precursor of PI(4,5)P2, and its inhibitors might impact the synthesis and levels of PI(4,5)P2, thereby affecting the regulatory role of PIP4K2A. Gallstone medications: Certain drugs used to dissolve gallstones or prevent their formation may affect cholesterol transport and the expression of related regulatory factors, including PIP4K2A. Cell signaling pathway modulators: Medications or therapeutic approaches might influence the expression and function of PIP4K2A by regulating cell signaling pathways, such as the phosphoinositide signaling pathway. Cholesterol-regulating drugs: Some medications aimed at regulating cholesterol metabolism and transport could impact the expression of PIP4K2A since PIP4K2A is involved in regulating LMPC and cholesterol transport. Metabolic-regulating medications: Drugs like insulin may affect the expression and function of PIP4K2A by modulating cellular metabolic pathways, thereby influencing LMPC and cholesterol transport. Gene therapy: Treatment methods targeting the PIP4K2A gene could directly impact its expression levels or function, for example, by regulating the expression of PIP4K2A through gene editing technologies.

RAB1A is a small GTPase well-known for its role in vesicle transport [47]. It has been reported that RAB1A mediates the conversion of proinsulin to insulin. In our study, we examined glucose-stimulated insulin secretion in INS-1E cells that possess RAB1A functionality and observed a reduction in insulin secretion in rat pancreatic cells [48]. These findings suggest that the RAB1A gene may have an impact on the development of diabetes. Here is a brief summary of how existing therapeutic methods can influence the expression of RAB1A. Vesicle transport regulators: Medications or treatments can directly modulate the vesicle transport pathway, affecting the expression or activity of RAB1A and consequently impacting intracellular substance transport and secretion. Antiviral drugs: Certain antiviral medications may influence intracellular vesicle transport, potentially inhibiting virus replication and spread by affecting the function of RAB1A. Cell division inhibitors: Some drugs might affect vesicle transport during the cell division process, thus influencing the role of RAB1A in cell division. Cell apoptosis modulators: Medications or treatment methods could indirectly impact the expression of RAB1A by regulating the cell apoptosis pathway, as cell apoptosis may involve the regulation of vesicle transport pathways. Metabolic-regulating medications: Drugs could affect cellular metabolic pathways, consequently influencing the expression or activity of RAB1A due to the close relationship between cellular metabolism and vesicle transport. Gene therapy: Therapeutic approaches targeting the RAB1A gene could directly alter its expression levels or function, such as through gene editing techniques for regulating RAB1A expression.

PTPN9 enhances glucose uptake in mature C2C12 myocytes and 3T3-L1 white adipocytes by stimulating the Akt pathway or adenosine monophosphate–activated protein kinase (AMPK) [49]. Studies have identified PTPN9 as a potential target for antidiabetic interventions and its multiple target natural products [50]. Some therapeutic interventions can impact its expression. Insulin regulators: Insulin, a pivotal hormone in regulating glucose metabolism, and its analogues may influence the expression and functionality of PTPN9 by modulating the Akt pathway, thereby affecting glucose absorption. AMPK activators: Agents activating AMPK could directly affect the activity of PTPN9 through the AMPK pathway, consequently impacting glucose metabolism and absorption. Exercise therapy: Moderate physical activity can influence glucose metabolism and insulin sensitivity through various pathways, potentially modulating glucose absorption by regulating the expression of PTPN9 and the AMPK pathway. Metabolic modulators: Certain medications might alter cellular metabolic pathways, such as the glucose metabolism pathway, affecting the expression or function of PTPN9 and consequently influencing glucose absorption. Nutritional interventions: Nutrient components in the diet can influence glucose metabolism and related signaling pathways, potentially regulating glucose absorption by modulating the expression of PTPN9. Gene therapy: Therapeutic approaches targeting the PTPN9 gene could directly impact its expression levels or function, for instance, through genetic editing techniques to regulate PTPN9 expression.

SNUPN, encoding SNURPORTIN-1, plays a central role in the nuclear import of small nuclear ribonucleoprotein particles in spliceosomes [51]. However, its physiological functions are still not fully explored. To date, no studies have demonstrated an association between this gene and various diseases [52]. Our research results indicate that the SNUPN gene may be a promising therapeutic target for antidiabetic interventions. The following are therapeutic methods that influence its expression. RNA interference (RNAi) therapy: RNAi technology can be utilized to target and silence the expression of the SNUPN gene, thereby impacting the protein levels of SNUPN. Gene editing: Gene editing technologies such as CRISPR-Cas9 can be employed to directly edit the SNUPN gene, thereby affecting its expression levels or functionality. Protein degradation regulators: Regulators of protein degradation may affect the stability and degradation rate of SNUPN, consequently influencing its expression levels. Posttranslational protein modification regulators: Medications or therapeutic approaches might influence the function and expression of SNUPN by modulating posttranslational protein modification pathways such as phosphorylation or ubiquitination. Cellular signaling pathway regulators: Medications or therapeutic methods could impact the expression or functionality of SNUPN by modulating cellular signaling pathways, including those associated with nuclear import pathways. Protein transport regulators: Drugs could alter intracellular protein transport pathways, thereby affecting the role and expression of SNUPN in nuclear import.

The integration of pQTL with GWAS data on CVDs has provided an insightful MR analysis at the proteomic level. This analysis has substantiated the causal relationship between plasma proteins and the risk of CVDs, including CHF, IS, and T2DM. These findings offer novel insights for the treatment of CVDs. This study possesses notable strengths in its methodology. Firstly, the implementation of a MR design minimizes potential confounding factors and biases arising from reverse causality. Additionally, the inclusion of cis-pQTLs (cis-pQTLs > trans-pQTLs > eQTLs) enhances the level of evidence, followed by gene colocalization analysis, which improves statistical power. Gene colocalization analysis has proven to be a robust tool for uncovering pleiotropic effects of certain loci on multiple traits, thereby enhancing the credibility of the research findings. Moreover, the utilization of large-scale GWAS increases the ability to detect associations ranging from mild to moderate. Another strength lies in the restriction of the analysis to individuals of European ancestry, thereby minimizing population stratification biases. Lastly, the comprehensive phenotypic association analysis enables a more in-depth exploration of potential drug target implications and their associated side effects.

There are several limitations that should be acknowledged in our analysis. Firstly, it relies on strict core assumptions. While we carefully selected SNPs at a genome-wide significance level and *F*-statistics indicate strong genetic associations with plasma protein levels, it is important to acknowledge that our research findings may be influenced by weak instrument bias. Secondly, although gene colocalization analysis helps alleviate biases arising from LD, horizontal pleiotropy may still not be fully minimized. Additionally, due to resource limitations, we were unable to perform population-stratified analyses. Therefore, it is worth noting that if there are significant differences in allele frequencies between different populations, our conclusions may be compromised, as this study is solely based on individuals of European ancestry. Lastly, despite the inclusion of a large number of plasma proteins in this study, it is possible that we inadvertently overlooked important proteins lacking genetic instruments. Further investigation is warranted to explore the risk prediction capabilities of these identified positive proteins.

## 5. Conclusion

This study investigated the causal relationships between nine plasma proteins (AZGP1, ALDH2, APOE, JAG1, MCL1, PIP4K2A, RAB1A, PTPN9, SNUPN) and CVDs, providing new targets for the treatment of such conditions. However, it is important to note that our MR study is observational in nature, and further research is needed to validate the potential causal relationships we have identified. We look forward to future studies that delve deeper into these drug targets, their potential therapeutic approaches, and their impact on the treatment of CVDs.

## Figures and Tables

**Table 1:** Data sources for outcome indicators used in the MR analyses.

**Phenotype**	**ID**	**Sample size**	**Number of SNPs**	**Population**	**Sources**
Atrial fibrillation	ebi-a-GCST90038689	484,598	9,587,836	European	UK Biobank
Coronary artery disease	ebi-a-GCST90013864	352,063	11,027,870	European	UK Biobank
Ischemic stroke	ebi-a-GCST90018864	484,121	24,174,314	European	UK Biobank and FinnGen
Type 2 diabetes	ebi-a-GCST90018926	490,089	24,167,560	European	UK Biobank and FinnGen
Venous thromboembolism	finn-b-I9_VTE	218,792	16,380,466	European	FinnGen
Peripheral artery disease	ebi-a-GCST90018890	483,078	24,186,090	European	UK Biobank and FinnGen
Chronic heart failure	ebi-a-GCST90018806	486,160	24,178,220	European	UK Biobank and FinnGen
Essential hypertension	ebi-a-GCST90038608	484,598	9,587,836	European	UK Biobank
Arterial stiffness	ebi-a-GCST008403	127,121	9,342,319	European	UK Biobank

**Table 2:** Results of gene colocalization analysis between plasma proteins and CVDs.

**Plasma protein**	**PPH0**	**PPH1**	**PPH2**	**PPH3**	**PPH4**	**PPH3 + PPH4**
Results of gene colocalization analysis between plasma proteins and CAD						
LAYN	0.659228078	0.338805852	3.82e − 06	0	0.001962251	0.001962251
KPNA2	0.997172149	0.002728981	3.20e − 05	2.07e − 08	6.69e − 05	6.68757e − 05
GCKR	0.997752117	0.002229193	5.78e − 06	0	1.29e − 05	1.29108e − 05
MSMP	2.13E-08	0.994591833	1.28e − 12	5.43e − 05	0.005353811	0.005408146
ADH1B	0.99727104	0.002541303	0.000135123	2.92e − 07	5.22e − 05	5.25348e − 05
A4GNT	0.999458933	0.000502011	3.45e − 05	1.27e − 08	4.57e − 06	4.58e − 06
COL6A3	0.999522233	0.000441503	3.33e − 05	1.17e − 08	2.98e − 06	2.99665e − 06
KLC1	0.999980856	5.26e − 06	1.38e − 05	0	7.27e − 08	7.267e − 08
CALB2	0.999743572	0.000215073	3.90e − 05	6.01e − 09	2.37e − 06	2.38062e − 06
Results of gene colocalization analysis between plasma proteins and CHF						
PLG	1.6959e − 140	0.656256797	8.1748e − 141	0.316314723	0.02742848	0.343743203
AZGP1	4.2623e − 153	0.074058761	1.505e − 153	0.025248656	0.900692583	0.925941239
Results of gene colocalization analysis between plasma proteins and IS						
PELO	0.001116717	0.989644667	8.08e − 06	0.00715642	0.002074118	0.009230539
ALDH2	7.81438e − 14	0.000631233	1.23694e − 10	0.99918311	0.000185657	0.999368767
Results of gene colocalization analysis between plasma proteins and T2DM						
CCL27	3.96e − 05	0.886318279	1.75e − 06	0.039085229	0.074555126	0.113640354
APOE	5.999e − 173	1.49958e − 09	4.0004e − 164	0.999999996	2.54259e − 09	0.999999999
BMP7	3.75e − 33	0.903258315	1.45e − 34	0.034948489	0.061793196	0.096741685
ANGPTL4	1.6051e − 157	0.947074511	7.1182e − 159	0.041990199	0.01093529	0.052925489
JAG1	1.38e − 64	3.36e − 05	4.08e − 60	0.996382303	0.003584098	0.999966401
FGFR1	3.0845e − 103	0.903258315	1.1956e − 104	0.034948489	0.061793196	0.096741685
MLN	4.60e − 301	0.233519777	1.49e − 300	0.75669032	0.009789903	0.766480223
MSMP	5.13e − 46	0.952701402	1.93e − 47	0.035807006	0.011491592	0.047298598
MCL1	1.75e − 73	0.052793716	2.73e − 73	0.081544742	0.865661541	0.947206284
11109_56_SVEP1	1.96e − 221	0.344336491	1.12e − 222	0.018992519	0.63667099	0.655663509
11178_21_SVEP1	2.52e − 258	0.344336491	1.44e − 259	0.018992519	0.63667099	0.655663509
PTPN9	7.68e − 16	2.04e − 06	3.76e − 10	0.999985509	1.24e − 05	0.999997958
PIP4K2A	1.00e − 39	0.026009785	1.60e − 39	0.040631108	0.933359108	0.973990215
RFK	9.02e − 97	0.887782176	4.28e − 98	0.042087971	0.070129853	0.112217824
HEXIM2	6.50e − 20	0.275245245	1.40e − 19	0.594445453	0.130309302	0.724754755
SNUPN	9.89e − 44	9.89e − 07	3.72e − 38	0.371491393	0.628507617	0.999999011
VAT1	4.71e − 32	0.956583166	1.40e − 33	0.028357296	0.015059537	0.043416834
ALDH2	1.20e − 10	0.967593852	1.90e − 12	0.015294116	0.017112033	0.032406148
COMT	2.06e − 163	0.834295038	5.30e − 165	0.021291686	0.144413276	0.165704962
RAB1A	2.72e − 09	5.10e − 05	5.33e − 05	0.999744512	0.000151108	0.999895619

## Data Availability

The data that support the findings of this study are available in the supporting information of this article.

## Supporting Information

Supporting Information 1 Supporting Information 1: SNP information involved in the MR analysis.

Supporting Information 2 Supporting Information 2: The results of MR analysis of plasma proteins and CVDs.

Supporting Information 3 Supporting Information 3: Result of PheWAS analysis of associations between nine CVD-associated plasma proteins and other disease outcomes.
